# The Anti-Inflammatory Effect of Zhibaidihuang Decoction on Recurrent Oral Ulcer with Sirt1 as the Key Regulatory Target

**DOI:** 10.1155/2021/8886699

**Published:** 2021-05-03

**Authors:** Yajie Shao, Bin Ding, Jinjun Ji, Shanshan Lei, Yu Dong, Yongsheng Fan, Weihong Ge, Li Xu

**Affiliations:** ^1^College of Basic Medicine, Zhejiang Chinese Medical University, Hangzhou, Zhejiang, China; ^2^College of Life Science, Zhejiang Chinese Medical University, Hangzhou, Zhejiang, China; ^3^Zhejiang Academy of Traditional Chinese Medicine, Hangzhou, Zhejiang, China; ^4^First Clinical Medical College, Zhejiang Chinese Medical University, Hangzhou, Zhejiang, China; ^5^College of Pharmaceutical Science, Zhejiang Chinese Medical University, Hangzhou, Zhejiang, China

## Abstract

The syndrome of ROU is generally manifested as obvious pain, redness, and swelling of local ulceration area, accompanied by flushed face, red eyes, sore throat, and swollen gums. Traditional Chinese medicine (TCM) doctors believe that “yin deficiency” is one causative factor of ROU. Zhibaidihuang decoction (ZBDHD) is a prescriptively developed receipt, where *Anemarrhena asphodeloides* and *Phellodendri amurensis* Cortex are added in the original Liuweidihuang decoction. It is generally used for “yin deficiency” treatment. It can effectively reduce the recurrence of oral ulcers and release the severity of the disease. However, the mechanism of this activity remains to be elucidated. In this study, we found that ZBDHD has a certain therapeutic effect on the pathological changes of oral mucosa. Furthermore, the results of serum metabolomics showed ZBDHD influenced the synthesis and metabolism of certain fatty acids. The results of western blot, immunochemical, and immunofluorescence staining indicate that ZBDHD could increase the expression of Sirt1 and Foxp3 and suppress the expression and acetylation of NF-*κ*B in oral mucosa cells. By screening active ingredients in ZBDHD, we found berberine, as well as other compounds, presenting high fitness of the Sirt1 reactive centre. Therefore, it is possible that ZBDHD can regulate the Sirt1-NF-*κ*B pathway to improve fatty acids metabolism in the body, thereby achieving the effect of treating ROU.

## 1. Introduction

Recurrent oral ulcer (ROU) is one of the most common oral mucosal diseases and its incidence rate is 30% [1]. Come so far, the mechanism is still unclear; most studies indicate that ROU is associated with immune inflammation [2]. It was considered that the ROU included five stages including initiation, primary damage response, expanded damage response, ulcer formation, and repair, while inflammatory response was throughout the process. The clinical research showed that CD4^+^, CD25^+^, and Foxp3^+^ were significantly reduced [3]. On the contrary, IL-6 and TNF-*α* concentration was significantly increased in peripheral blood of recurrent ulcers patients [4]. Animal experiments showed that the expression of NF-*κ*B p65 [5] and TNF-*α* significantly increased in recurrent oral ulcer model rats [6]. These previous reports demonstrated that ROU is related to immune response and inflammatory response.

The Chinese medicine theory mentions that the unevenness of “yin” (cold) and “yang” (hot, fire) in the human body could result in diseases. TCM doctors considered that excess “yang” (fire) is an important factor in causing ROU in clinic [7]. The excess fire could be due to “yinxu,” which means “yin deficiency”. Fuzi (Radix Praeparata *Aconitum carmichaelii* Debx), Ganjiang (Rhizoma *Zingiber officinale*), and Rougui (Cortex *Cinnamomum cassia*) decoction (AZC) is one of the prescriptions for hyperthermia, used to treat “cold” syndrome. However, overdose of AZC could break the balance between “yin” and “yang,” which produces heat and inflammation [8]. Previous work indicated that AZC could induce excess of “fire” to cause oral ulcer [9, 10].

Zhibaidihuang decoction (ZBDHD), a traditional Chinese herbal receipt, is usually used to cure ROU and hyperactivity of “fire” due by “yin” deficiency in clinic [11, 12]. The receipt was composed of eight herbs including Zhimu (Rhizoma *Anemarrhena asphodeloides*), Huangbai (*Phellodendri amurensis* Cortex), Shudihuang (Radix *Rehmanniae Preparata*), Shanzhuyu (Fructus *Cornus officinalis*), Shanyao (Radix *Dioscorea opposita*), Fuling (Sclerotium of *Poria cocos*), Mudanpi (Cortex *Paeonia suffruticosa*), and Zexie (Rhizoma *Alisma orientale*). In this prescription, Fuling, Mudanpi, and Zexie have capability of eliminating excess “yang” (fire). And Huangbai and Zhimu show activity on enhancing the efficiency. The active components in this receipt are very complex. This is due to the complex mechanisms of ZBDHD treatment. It was identified with modern biological method that AZC can influence the AMPK/Sirt1 pathway [10], as well as expression of NF-*κ*B, one of inflammatory response cytokines [13]. Previous studies proved that Sirt1 was correlated to cell metabolism [14, 15]. And Sirt1 is the key cytokine, which regulates the transcription of NF-*κ*B [16, 17]. Therefore, in this research, we evaluated the regulation mechanism of ZBDHD treatment on oral mucosa through Sirt1/NF-*κ*B pathway with a AZC treated ROU rat model.

## 2. Materials

### 2.1. Materials and Regents

Freund's complete adjuvant (8642852) was purchased from MP Biomedicals (California, USA). The specific antibodies against Sirt1 (sc-74465), NF-*κ*B p65 (sc-372), Stat3 (sc-482), Foxp3 (sc-28705), acetyl lysine (sc-284922), and TNF-*α* (sc-4261) were all purchased from Santa Cruz Biotechnology (Texas, USA). The secondary antibodies, Goat anti Rabbit IRDYE @ 800 CW (C10324-01), and Goat anti Mouse IRDYE @ 680 (C40312-02) were all purchased from LICOR (Nebraska, USA). Fetal bovine serum and RPMI1640 cell culture medium were obtained from GENOM (Hangzhou, China). The primers used in this study were synthesized by Bioengineering (Shanghai, China).

### 2.2. Animals

Fifty healthy (180∼200 g) female SD rats were purchased from Shanghai SLAC Lab. Animal Ltd. (Shanghai, China) and housed in laboratory animal research centre of Zhejiang Chinese Medical university. All animal experiments have passed the ethics committee with accepted Nr. ZSLL-2016-116.

### 2.3. Decoction Preparation

All of the herbs were purchased from Zhejiang Province Famous Doctor Pavilion and Zhejiang Chinese Medical University Medical Slicing Factory. The preparation of AZC decoction refers to our previous publication [8]. ZBDHD preparation was also described previously [13] and was diluted to specific concentration for experiments. And the decoction concentration in this work presented as milli gram herbal mixture per milli liter.

### 2.4. Animal Treatment

In this experiment, six rats (control group, Ct), were given distilled water. For model preparation, the oral mucosa from twenty healthy SD rats was mixed with complete Freund's adjuvant (1 : 1 *v*/*v*) and sterile-filtrated with a sterile filter (0.22 *μ*m diameter). Twenty-four rats were subcutaneously injected 0.1 mL this mucosa emulsion on both sides of back once a week. And rats were internally orally injected 0.1 mL mucosa emulsion, also. Simultaneously, rats were given 4 mL 1 g/mL AZC by intragastric administration (i.g.) every day for 4 weeks. For ZBDHD protective efficiency evaluation, these twenty-four rats were separated into four groups. One group (model group, AZC) was given 4 mL/day distilled water by intragastric administration (i.g.). Simultaneously, the other three groups were treated with 4 mL/day high dose (2 g/mL), middle dose (1 g/mL), and low dose (0.5 g/mL) ZBDHD by i.g. for a week, respectively, which were marked as ZBDHD-H, ZBDHD-M, and ZBDHD-L.

### 2.5. Histological Study

The 10% formalin solution fixed oral mucosa (of each rat in different groups) was embedded in paraffin which was cut and stained with Hematoxylin & Eosin (H&E) stain for electron microscope (Carl Zeiss, Germany) observation.

### 2.6. Immunohistochemistry and Immunofluorescence Image

The paraffin-embedded sample was cut into thin sections (4-5 *μ*m) and sealed in 3% H_2_O_2_ at room temperature to inactivating enzymes. Then, the sections were boiled in 10 mM sodium citrate buffer (pH 6.0) for 10 min and cooled at room temperature. For immunohistochemistry analysis, the sections were hybridized with primary antibodies against acetyl lysine (1 : 200), Sirt1 (1 : 200), NF-*κ*B (1 : 200), and TNF-*α* (1 : 200), respectively, overnight at 4°C. The primary antibodies were detected with fluorescence labelled corresponding secondary antibody. Simultaneously, API staining was performed to stain the nuclei. More details were described in previous studies [9, 18].

### 2.7. Serum Preparation and Metabolites Analysis

#### 2.7.1. Serum Preparation

The blood samples were stated at 37°C for one hour and centrifugated at 4000 rpm for 10 min. 150 *μ*L serum was pipetted and vortex mixed with 450 *μ*L acetonitrile to precipitated proteins. The supernatant was diluted 1 : 1 with deionized water and filtered with a 0.22 *μ*m nylon filter.

#### 2.7.2. HPLC-QTOF/MS Analysis

5 *µ*L filtered serum samples were loaded into a Waters Acquity UPLC C18 column (1.8 *µ*m, 2.1 × 50 mm), with Agilent 1260 HPLC/6520 QTOF-MS instrument (Agilent, Milford, MA, USA). The mobile phase consisted of 95% solvent A (0.1% (*v*/*v*) formic acid) and 5% solvent B (0.1% (*v*/*v*) formic acid acetonitrile solution). The optima gradient eluate programme was as follows: 0–4 min, 95% A; 4–17 min, 90% A to 55% A; 17–22 min, 55% A to 5% A. The parameters of electrospray ionization (ESI+) mass spectrum were drying gas (N_2_) flow rate, 10 L/min; gas temperature, 350°C; scan range, m/z 50∼1000; capillary voltage, 4000 V; fragmentation voltage, 180 V; cone voltage, 60 V; atomizing air pressure, 310 kPa. The acquisition rate was 2 spectra/s.

### 2.8. Western Blot

The expressions of signalling molecules, Sirt1, NF-*κ*B p65, Stat3, and Foxp3 in oral mucosa cell from different group rats were comparatively identified with western blot. The oral mucosa was sheared in lysate buffer by tissue homogenizer, and cell debris was discharged by centrifugation. The protein concentration in cell lysate was quantified with Bradford. 20–30 *μ*g proteins were mixed with loading buffer and separated with an SDS-PAGE gel. For western blot analysis, the proteins in gel were electrically transferred on a nitrocellulose filter membrane at 80 V for 2 hours in a transfer buffer system (3.03 g Tris + 14.4 g glycine + 200 mL methanol in ddH_2_O to a final volume of 1000 mL). Before hybridization, the membrane was cut according to the mass of detected proteins and then incubated with antibodies specific for *β*-actin, Sirt1, NF-*κ*B p65, Stat3, or Foxp3 separately at 4°C overnight. On the next day, membranes were washed and incubated with the respect secondary antibody for 2 hours. The blots were exposed and scanned using an Odyssey Infrared Imaging system (LI-COR Biosciences), and the blots were quantified using the Odyssey Infrared Scanning system software.

### 2.9. Identification of Sirt1 Regulator(s) in ZBDHD

All chemical compounds in ZBDHD were first selected from the TCMSP database (http://lsp.nwu.edu.cn/tcmsp.php) [19]. Three in silico ADME models, OB (oral bioavailability, threshold ≥ 30%), DL (drug-likeness, threshold ≥ 30%), and Caco-2 (Caco-2 permeability, threshold ≥ 30%), were used, respectively. The inhibitory affinity of eight major components with human Sirt1 was validated by molecular docking using AutoDock4.2.0 software [20].

### 2.10. Statistical Analysis

All the data in this study were expressed as mean ± standard deviation (SD). And *t*-test was used to determine the differences between control and treatment groups with SPSS 15.0. The statistically significant difference was considered at level *P* < 0.05.

## 3. Results

### 3.1. Pathological Changes of Oral Mucosa Induced by AZC and Proactive Effect of ZBDHD

**Figure 1:**
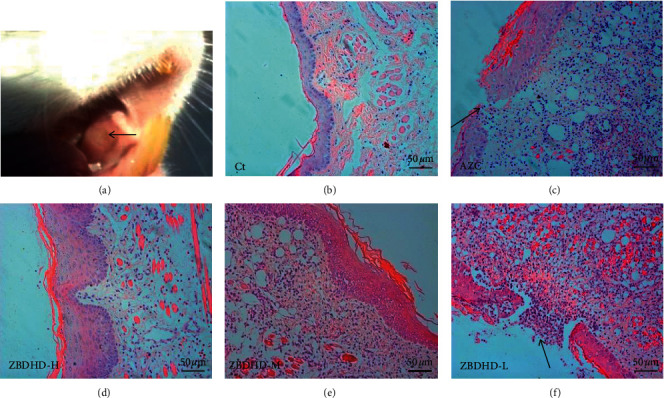
AZC model and protective effect on oral mucosa of ZBDHD treatments. (a) Ulcer (indicated by the black arrow) in oral mucosa of AZC model rat. (b–f) Histopathologic study of oral mucosa stained by hematoxylin and eosin (H&E) (x200); Ct, AZC, ZBDHD-H, ZBDHD-M, and ZBDHD-L indicated control, AZC model, high-dose ZBDHD treatment (2 g/mL), middle-dose ZBDHD treatment (1 g/mL), and low-dose ZBDHD treatment (0.5 g/mL), respectively.

In 4 weeks, the oral mucosa of the model group exhibited irritation and inflammation, which is shown in Figure 1(a) and indicated by a black arrow. The aberrant hyperplastic epithelium and infiltrating of inflammatory cells could be observed in H&E strained oral mucosa of AZC model rats (Figure 1(c)), but not in control group rats (Figure 1(b)). In comparison with that of rats in AZC model group, oral mucosa of ZBDHD (high dose and middle dose) (Figures 1(d) and 1(e)) treated rats showed clear cell structure and improved of inflammation, obviously. However, no positive effects could be observed in ZBDHD (low dose) group (Figure 1(f)). The changes of body weights during the last 7 days treatment were monitored (Table 1). Although those of the ZBDHD treated rats were larger than those of model rats, no significant differences were observed.

### 3.2. ZBDHD Influences the Metabolites Variation

**Figure 2:**
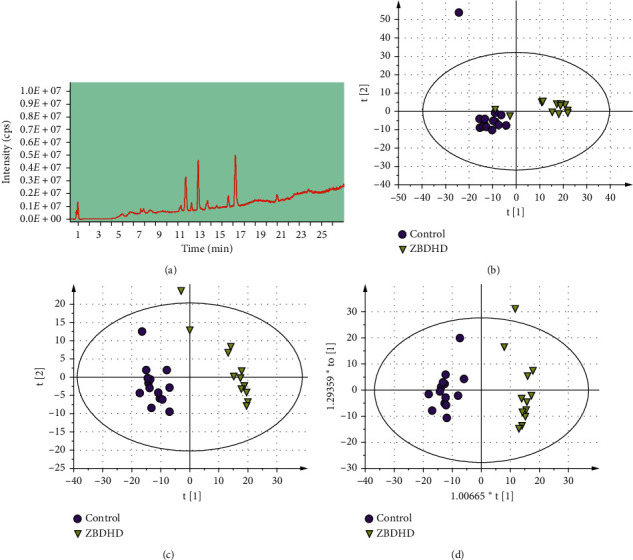
HPLC-QTOF/MS analysis of the serum from the ZBDHD-H treated rat. (a) HPLC-based analysis of serum. (b) PCA score plot of serum samples between the rats in ZBDHD and those in control group (R2X = 0.42, Q2 = 0.0913); (c) PLS-DA score plot between the two groups (R2X = 0.281, R2Y = 0.953, Q2 = 0.777); and (d) OPLS-DA score plot between the two groups (R2X = 0.281, R2Y = 0.953, Q2 = 0.772): control group and ZBDHD group.

As a part of system biology, metabolomics is a new discipline which can demonstrate the curative effect of TCM therapy. In this study, metabolic profiles of serum samples from rats in control and ZBDHD-H (Figure 2(a)) groups were analyzed by HPLC-QTOF/MS. The data were discriminated with the principal component analysis (PCA) (Figure 2(b)), partial least squares discriminant analysis (PLS-DA) (Figure 2(c)), and the orthogonal partial least squares-discriminate analysis (OPLS-DA) (Figure 2(d)). The metabolites were considered as potential biomarkers responsible for the metabolic profile, when *P* value of *t*-test <0.05 and VIP >1. In comparison with those of control group, phytosphingosine, palmitic amide, and MG (0 : 0/16 : 0/0 : 0) in serum of middle dose ZBDHD treated rats were significantly decreased. On the contrary, *α*-Linolenic Acid, LysoPC (20 : 4 (5Z, 8Z, 11Z, 14Z), LysoPC (20 : 3 (5Z, 8Z, 11Z), and LysoPE (0 : 0/20 : 0) were significantly increased (Table 2). Screening KEGG database [9], these metabolites were related to fatty acid biosynthesis, linoleic acid, and glycerophospholipid metabolism (Table S1). And Sirt1 could be one of the key regulatory cytokines.

### 3.3. The Effect of ZBDHD on Sirt1/NF-*κ*B Pathway in Oral Mucosa

**Figure 3:**
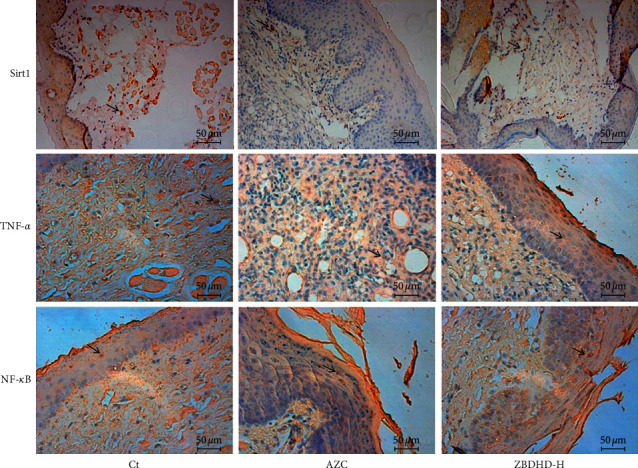
IHC analyses of expression of Sirt1, NF-*κ*B, and TNF-*α* in oral mucosa. The expressed Sirt1, NF-*κ*B p65, and TNF-*α* in oral mucosa of Ct, AZC, and ZBDHD-H rats were immunohistochemically (IHC) analyzed and observed under the microscope (x200). The positive cells were pointed out by arrows.

**Figure 4:**
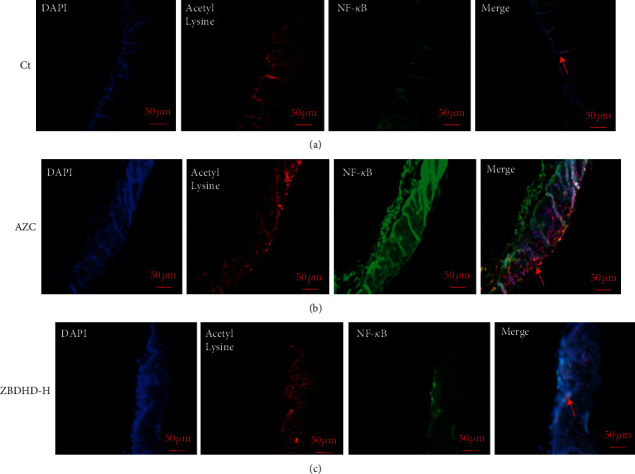
IFC demonstration of deacetylation NF-*κ*B in oral mucosa.

**Figure 5:**
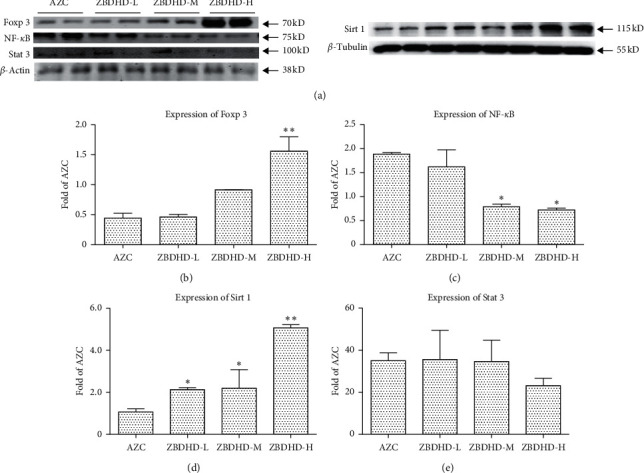
Expression of Sirt1, Foxp3, NF-*κ*B, and Stat3 in oral mucosa from different group rats. (a) Western blot analyses of Sirt1, Foxp3, NF-*κ*B, and Stat3 in oral mucosa. (b–e) The expression of Sirt1, Foxp3, NF-*κ*B, and Stat3 in ZBDHD-L, ZBDHD-M, and ZBDHD-H versus AZC in oral mucosa; Data are mean ± SD (*n* = 3). ^*∗*^*P* < 0.05, ^*∗∗*^*P* < 0.01 vs AZC group.

Sirt1 is a protein deacetylase that interferes with the NF-*κ*B signaling pathway, which regulates cell anti-inflammatory function. Therefore, the expression of cytokines, Sirt1, NF-*κ*B, and TNF-*α*, in oral mucosa of rats in different groups, was immunohistochemically imaged in brown (Figure 3). In the cell of model rats, the expressed Sirt1 was obviously less than those of control rats. On the contrary, the expressed NF-*κ*B and TNF-*α* were more than those of control group. The expression of Sirt1, NF-*κ*B, and TNF-*α* in oral mucosa cells of rat was ameliorated by ZBDHD treatment. Simultaneously, the location of Sirt1 (red stained) and deacetylated NF-*κ*B (green stained) in cells was studied with immunofluorescence detection (Figure 4). The yellow signal could be obtained in merged photos. In comparison, in the model group (Figure 4 Merge) more yellow signal could be observed than in the other two groups. To confirm this result, the expression of Sirt1 and NF-*κ*B in cells was studied by western blot, as well as Foxp3 and Stat3. The high dose ZBDHD treatment (2 g/mL) could inhibit the expression of NF-*κ*B and stimulate the expression of Sirt1, significantly (Figure 5). In addition, the expression of Foxp3 was also influenced by the treatment in a dose-dependent manner. However, the expression of Stat3 was stimulated by ZBDHD but not significantly.

The position of cell nucleus, acetyl lysine, and NF-*κ*B was, respectively, hybridized with blue, red, and green fluorescein labeled specific antibodies. The yellow spot, in merged image, indicated the co-location of acetyl lysine and NF-*κ*B (red arrow marked).

### 3.4. Screening Active Ingredients in ZBDHD Which Docked the Reactive Centre in Sirt1

Bioinformatic technology was engaged in identifying the active substances in ZBDHD. Based on TCMSP database (http://lsp.nwu.edu.cn/tcmsp.php), there were 729 kinds of known ingredients, including 81 kinds in Rhizoma *Anemarrhena asphodeloides*, 141 kinds in *Phellodendri amurensis* Cortex, 76 in Radix *Rehmanniae preparata*, 226 in Fructus *Cornus officinalis*, 71 in Radix *Dioscorea opposita*, 34 in Sclerotium of *Poria cocos*, 55 in Cortex *Paeonia suffruticosa*, and 46 in Rhizoma *Alisma orientale* in eight herbs of ZBDHD. In silico ADME molecular docking model (OB ≥ 30%, DL ≥ 0.18 and Caco-2 ≥ 0.4) was utilized to virtually screen these ingredients, in which 78 compounds represent the potential docking properties on Sirt1 (Table S2). The interaction specification was evaluated by affinity score. And eight of the most sensitive candidates are shown in Table 3, according to their affinity score.

**Figure 6:**
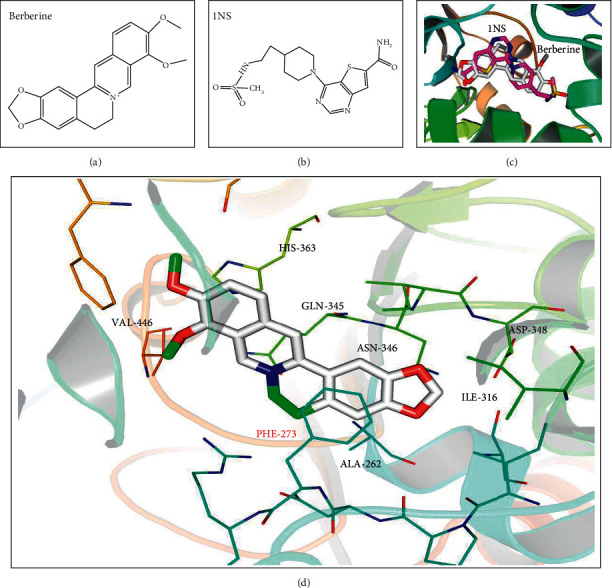
Docking of berberine in active centre of Sirt1. (a) Chemical structure of berberine. (b) Chemical structure of INS. (c) Docking of berberine and INS with Sirt1. (d) Berberine interacts with the amino acid residues in active centre of Sirt1.

Berberine is one well-known component in *Phellodendri amurensis* Cortex. We hypothesized that berberine (Figure 6(a)) can dock in the reactive centre of Sirt1. A docking model was generated with the well-known Sirt1 inhibitor, 1 NS (4-(4-[2-[[methylsulfonyl] amino] ethyl] piperidin-1-yl)thieno (3, 2-d)pyrimidine-6-carboxamide) (Figure 6(b)). The superimposition of 1 NS and berberine is shown in Figure 6(c). The active centre parameters of berberine in Sirt1 were *X* = 5.641; *Y* = 41.417; *Z* = −1.615, which was calculated with the Autodock 4.2.0 software. The 3D structure of Sirt1-berberine complex was figured out by PyMol software (Figure 6(d)). The binding energy of Sirt1 and berberine was −9.9 kcal/mol, which indicated a good interaction result. From the complex, amino acid residues of PHE-273 in Sirt1 and benzene in berberine could form the *π*-*π* conjugated.

## 4. Discussion and Conclusion

Herbal medicines have been used to treat various diseases for many centuries, in Chinese society. Zhibaidihuang decoction (ZBDHD), a poly herbal formula, has been used to treat syndrome of hyperactivity of “yang” (fire) due to “yin” deficiency for thousands of years [21]. Recurrent oral ulcers are a typical symptom of hyperactivity of “fire” due to “yin” deficiency [22]. In modern medicine, oral ulcer is a kind of inflammatory diseases, which occurs due to a damage in epithelium induced by many certain factors, such as hormonal changes [23], lack of essential vitamins [24], and disorder of metabolism which could induce oral ulcer [25]. It is validated by clinical trial that ZBDHD treatment could promote the ulcer healing [26] and improve the fitness of recurrent oral ulcer significantly [13].

Our previous research shows the extracts of AZC herbs may induce “fire” syndrome by influencing Treg cells and immunosuppressive cytokines [8]. In this research, AZC combined with immunological method induced immune dysfunction and oral ulcer which was similar to ROU clinical symptoms. Histopathological studies showed the cell inflammation infiltration and mucosal surface damage in model rats (Figures 1(a) and 1(c)). The protection capability of ZBDHD was studied in this research. Mucosal surface damage in model and low dose ZBDHD treated rats could be observed under microscope by H&E staining, but the rats treated with high and middle dose ZBDHD showed a sign of healing which was indicated the absence of damage (Figure 1). Simultaneously, the difference of metabolites between model group and high dose ZBDHD treatment group was evaluated by metabonomic study which indicated that ZBDHD treatment influenced synthesis and metabolite of some fatty acids, such as phytosphingosine and palmitic amide (Table 2). High dose ZBDHD treatment attenuated AZC induced distractive expression of cytokines, Sirt1, NF-*κ*B, and TNF-*α* (Figures 3 and 4). The results of our previous study also indicated that AZC can stimulate the expression of NF-*κ*B [13].The expression of cytokines in Sirt1 signaling pathway was quantitatively analyzed by western blot (Figure 5). Sirt1, which belongs to Sirtuins family, is involved in many human physiological processes, such as aging, DNA repair, cell apoptosis [27], and inflammatory response [28]. Sirt1 catalyzes the deacetylation of Lys 310 s in p65 sigma-subunit of NF-*κ*B [29], for suppression the regulation activity of NF-*κ*B [30]. To predict the phytochemistry and pharmaceutical mechanism of ZBDHD, we used the bioinformatic method to screen TCMSP database (http://lsp.nwu.edu.cn/tcmsp.php). A total of 78 ingredients present Sirt1 interaction potential. Eight of the 78 show relative high binding affinity with reactive centre of Sirt1 (Table 3). They are coptisine, berberine, sitosterol, diosgenin, mairin, anemarsaponin F_qt, trametenolic acid, and alisol B monoacetate. They have various pharmaceutical activities. For example, alisol B monoacetate has anti-tumor, anti-allergic, and inhibiting infection from hepatitis B virus [31]. Trametenolic acid can improve cerebral ischemia [32]. Anemarsaponin inhibits inflammation by inhibiting the phosphorylation of NF-*κ*B p38 [33]. Mairin, also called betulinic acid, has strong effects on anti-HIV, anti-inflammatory, anti-diabetic, and anti-microbial activities [34]. Diosgenin has anti-cancer, anti-diabetic, anti-coagulation, anti-thrombosis, anti-inflammatory, anti-viral, and anti-aging properties [35]. Sitosterol has a strong effect on decreasing blood fat and cholesterol [36]. Coptisine and berberine present higher score than others. Both ingredients are the structurally related isoquinoline alkaloids. But the results of previous studies indicated their differential functions. Coptisine, an antioxidant [37], could induce autophagy to exert anti-cancer effects [38]. No references indicated the anti-inflammatory activity of coptisine. In comparison, berberine was reported to have a strong effect on anti-inflammatory, previously [39]. As a powerful supplement with many benefits, berberine, one of the eight candidates, showed high binding affinity with the amino acid residues in active centre of Sirt1 (Figure 6). To our knowledge, it is the first time to evaluate the binding site of berberine on Sirt1.

In the present study, we used high dose “hot” herbs potions combined with immunological method to induce ulcer in mouth of model rats. In 14 days, the visible cavity in mucous membrane and decrease of body weight were observed in model rats, in comparison with the other groups. The phenomenon was identical to that in clinic [40]. Serum metabolomics analysis results showed that the metabolisms of some lipids, such as phytosphingosine, palmitic amide, and MG (0 : 0/16 : 0/0 : 0), were significantly inhibited by ZBDHD. Phytosphingosine [41], a phospholipid, is a major component of all biological membranes and sphingolipid metabolites. The palmitic amide is one of primary fatty acid amides, which are a kind of signaling molecules [42]. However, the function of palmitic amide is less known. MG (0 : 0/16 : 0/0 : 0) is a monoacylglyceride, consisting of a fatty acid chain covalently bonded to a glycerol molecule through an ester linkage. It is an intermediate in glycerophospholipid metabolism process [43]. On the contrary, ZBDHD treatment upregulated the serum concentrations of *α*-linolenic acid and LysoPC (20 : 4 (5Z, 8Z, 11Z, 14Z)), LysoPC (20 : 3 (5Z, 8Z, 11Z), and LysoPE (0 : 0/20 : 0). Both LysoPC (20 : 4 (5Z, 8Z, 11Z, 14Z)) and LysoPC (20 : 3 (5Z, 8Z, 11Z)) are lysophosphatidylchoine. LysoPE (0 : 0/20 : 0) are lysophosphatidylethanolamine. They are structural compounds in cell membrane at a low density. Thus, they are biomarkers of cell proliferation [44]. In addition, LysoPC and LysoPE are both post-injury marker metabolites [45]. LysoPC was a strong chemoattractant of T-lymphocytes, which can promote antibody formation and macrophages stimulus [46]. LysoPE is mobilizer of intracellular Ca^2+^ in some cell types [47, 48]. Calcium is one of the initial triggers in immune response for wound healing [49]. *α*-Linolenic acid served as substrate for unsaturated fatty acid synthesis, which showed anti-inflammatory effects [50]. Therefore, the results of metabolomics analysis indicated that the ZBDHD can reduce inflammation and promote the ulcer healing.

It had been reported that Sirt1, the silent information adjustment factor 2 related enzyme I, could suppress fatty acid metabolism [51]. In addition, Sirt1 can catalyze acetylation of transcription factor Forkhead Box P3 (Foxp3), an inhibitor of NF-*κ*B [52]. NF-*κ*B is an important pro-inflammatory factor and could influence the expression of inflammatory factors, such as TNF-*α*, IL-1*β*, and IL-8 [53]. Therefore, we hypothesized that Sirt1 could be one of the target factors of ZBDHD treatment.

## 5. Conclusion

**Figure 7:**
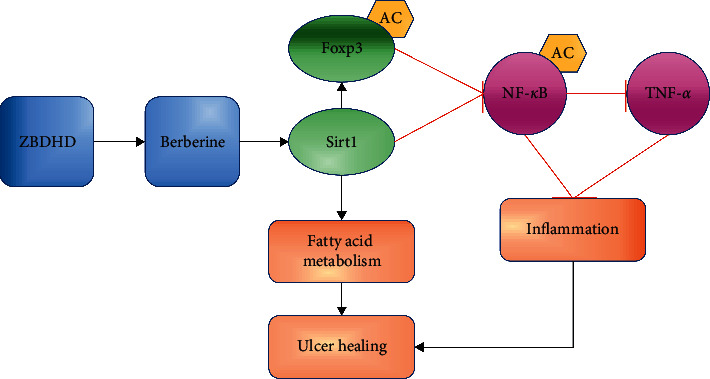
The schematic representation of ZBDHD regulation on ulcer healing and inflammation.

In present study, we demonstrated that ZBDHD treatment upregulated the expression of Sirt1 and attenuated the overexpression of TNF-*α*. These results confirmed that Sirt1 may be the main target of ZBDHD to reduce the mucosa inflammation. ZBDHD could improve the symptoms of oral ulcer in model rats. And the capability mechanism may be related to Sirt1-NF-*κ*B pathway, immune regulation, and metabolism regulation. And the berberine, one of the constituents in ZBDHD, is a key regulator of Sirt1 (Figure 7).

## Figures and Tables

**Table 1:** The body weight monitor in the last 7 days.

Group	Initial weight (g)	Final weight (g)	Increase (g)
Control group	255.83 ± 12.01	263.83 ± 11.20	8.00 ± 5.40
AZC group	236.83 ± 9.33	241.17 ± 7.91	4.33 ± 2.80
ZBDHD-H	239.33 ± 7.12	246.67 ± 9.79	7.33 ± 3.44
ZBDHD-M	233.17 ± 12.46	242.00 ± 16.19	8.83 ± 5.60
ZBDHD-L	228.17 ± 8.52	235.00 ± 8.88	6.83 ± 3.66

Note: the data are expressed as mean ± SD (*n* = 6).

**Table 2:** Differences metabolites between control and ZBDHD group in OPLS-DA analysis.

Name	VIP	RT	Mass	*T* test	Fold change (D/C)
Phytosphingosine	1.593	6.686	317.2931	0.034	−1.159
Palmitic amide	1.857	7.096	255.2558	0.012	−2.183
*α*-Linolenic acid	1.785	7.306	278.2241	0.016	0.923
LysoPC(20 : 4(5Z, 8Z, 11Z, 14Z))	1.813	10.710	543.3330	0.014	0.650
LysoPC (20 : 3(5Z, 8Z, 11Z))	1.570	11.839	545.3474	0.037	0.249
LysoPE (0 : 0/20 : 0)	2.086	13.097	509.3462	0.004	1.406
MG (0 : 0/16 : 0/0 : 0)	1.522	18.784	330.2751	0.044	−1.194

Note: fold change (D/C) is the logarithm of the ratio of the mean values of D to C (base 2). The positive sign indicates that D is increased relative to C and the negative sign indicates descent.

**Table 3:** Docking results of main active compounds in ZBDHD to Sirt1.

ID	Chemical name	Extraction code	Affinity score	Herb
M1458	Coptisine	4ZZI	10.6	*Phellodendri amurensis* Cortex
M1454	Berberine	4ZZI	9.9	*Phellodendri amurensis* Cortex
M0359	Sitosterol	4ZZI	7.4	Radix *Rehmanniae preparata*
M0546	Diosgenin	4ZZJ	6.1	Radix *Dioscorea opposita*
M0211	Mairin	4ZZJ	5.8	Cortex *Paeonia suffruticosa*
M4489	Anemarsaponin F_qt	4ZZJ	5.6	Rhizoma *Anemarrhena asphodeloides*
M0275	Trametenolic acid	4ZZJ	5.5	Sclerotium of *Poria cocos*
M0831	Alisol B monoacetate	4ZZJ	4.8	Rhizoma *Alisma orientale*

## Data Availability

All data used to support the findings of this study are included within the article, and these data can also be made accessible on website https://figshare.com/s/3ba0eaf51b3c2c78bc7b.

## Supplementary Materials

Supplementary Materials Table S1: related pathways of differences metabolites. Table S2: screening out 78 kinds of active compounds in Zhibaidihuang decoction.
