# Supplementary material for: The Anti-Inflammatory Effect of Zhibaidihuang Decoction on Recurrent Oral Ulcer with Sirt1 as the Key Regulatory Target
Source: Evid Based Complement Alternat Med. 2021 May 3;2021:8886699. doi: 10.1155/2021/8886699 (PMC8110403; doi:10.1155/2021/8886699)
Supplement: Supplementary Materials — Table S1: related pathways of differences metabolites. Table S2: screening out 78 kinds of active compounds in Zhibaidihuang decoction. [file 8886699.f1.docx]

**Table S1 Related pathways of differences metabolites**

| Pathway Name | Compound Name | P | FDR |
| --- | --- | --- | --- |
| Fatty acid biosynthesis | Palmitic acid,  Oleic Acid,  Myristic acid | 0.0049 | 0.19754 |
| Linoleic acid metabolism | Linoleic acid | 0.0421 | 1 |
| Glycerophospholipid metabolism | LysoPC(14:0)  LysoPC(16:0)  LysoPC(16:1(9Z))  LysoPC(18:1(11Z))  LysoPC(18:2(9Z,12Z))  LysoPC(20:0)  LysoPC(20:1(11Z))  LysoPC(20:4(5Z,8Z,11Z,14Z)) | 0.0003 | 0.02348 |

**Table S2 Screening out 78 kinds of active compounds in ZhibaiDihuang decoction**

| **ID** | **Compounds** | **MW** | **OB (%)** | **Caco-2** | **DL** | **Herb** |
| --- | --- | --- | --- | --- | --- | --- |
| MOL003773 | Mangiferolic acid | 442.75 | 36.16 | 0.66 | 0.84 | Rhizoma *Anemarrhena Asphodeloides* |
| MOL004373 | Anhydroicaritin | 368.41 | 45.41 | 0.72 | 0.44 | Rhizoma *Anemarrhena Asphodeloides* |
| MOL004489 | AnemarsaponinF_qt | 432.71 | 60.06 | 0.43 | 0.79 | Rhizoma *Anemarrhena Asphodeloides* |
| MOL004492 | Chrysanthemaxanthin | 584.96 | 38.72 | 0.51 | 0.58 | Rhizoma *Anemarrhena Asphodeloides* |
| MOL004514 | Timosaponin B III_qt | 416.71 | 35.26 | 0.42 | 0.87 | Rhizoma *Anemarrhena Asphodeloides* |
| MOL000449 | Stigmasterol | 412.77 | 43.83 | 1.44 | 0.76 | Rhizoma *Anemarrhena Asphodeloides* |
| MOL004540 | AnemarsaponinC_qt | 416.71 | 35.5 | 0.43 | 0.87 | Rhizoma *Anemarrhena Asphodeloides* |
| MOL000483 | (Z)-3-(4-hydroxy-3-Methoxy-phenyl)-N-[2-(4hydroxyphenyl)ethyl]acrylamide | 313.38 | 118.35 | 0.51 | 0.26 | Rhizoma *Anemarrhena Asphodeloides* |
| MOL000546 | Diosgenin | 414.69 | 80.88 | 0.82 | 0.81 | Rhizoma *Anemarrhena Asphodeloides* |
| MOL000631 | Coumaroyltyramine | 283.35 | 112.9 | 0.6 | 0.2 | Rhizoma *Anemarrhena Asphodeloides* |
| MOL001454 | Berberine | 336.39 | 36.86 | 1.24 | 0.78 | Cortex *Phellodendri Amurensis* |
| MOL001458 | Coptisine | 320.34 | 30.67 | 1.21 | 0.86 | Cortex *Phellodendri Amurensis* |
| MOL002643 | Delta 7-stigmastenol | 414.79 | 37.42 | 1.3 | 0.75 | Cortex *Phellodendri Amurensis* |
| MOL002644 | Phellopterin | 300.33 | 40.19 | 0.98 | 0.28 | Cortex *Phellodendri Amurensis* |
| MOL002651 | Dehydrotanshinone II A | 292.35 | 43.76 | 1.02 | 0.4 | Cortex *Phellodendri Amurensis* |
| MOL002652 | Delta7Dehydrosophoramine | 242.35 | 54.45 | 0.99 | 0.25 | Cortex *Phellodendri Amurensis* |
| MOL002656 | Dihydroniloticin | 458.8 | 36.43 | 0.65 | 0.81 | Cortex *Phellodendri Amurensis* |
| MOL002660 | Niloticin | 456.78 | 41.41 | 0.54 | 0.82 | Cortex *Phellodendri Amurensis* |
| MOL002662 | Rutaecarpine | 287.34 | 40.3 | 1.13 | 0.6 | Cortex *Phellodendri Amurensis* |
| MOL002663 | Skimmianin | 259.28 | 40.14 | 1.26 | 0.2 | Cortex *Phellodendri Amurensis* |
| MOL002666 | Chelerythrine | 332.37 | 34.18 | 1.24 | 0.78 | Cortex *Phellodendri Amurensis* |
| MOL000449 | Stigmasterol | 412.77 | 43.83 | 1.44 | 0.76 | Cortex *Phellodendri Amurensis* |
| MOL002668 | Worenine | 334.37 | 45.83 | 1.22 | 0.87 | Cortex *Phellodendri Amurensis* |
| MOL002670 | Cavidine | 353.45 | 35.64 | 1.08 | 0.81 | Cortex *Phellodendri Amurensis* |
| MOL002672 | Hericenone H | 580.88 | 39 | 0.8 | 0.63 | Cortex *Phellodendri Amurensis* |
| MOL000358 | Beta-sitosterol | 414.79 | 36.91 | 1.32 | 0.75 | Cortex *Phellodendri Amurensis* |
| MOL000785 | Palmatine | 352.44 | 64.6 | 1.33 | 0.65 | Cortex *Phellodendri Amurensis* |
| MOL000787 | Fumarine | 353.4 | 59.26 | 0.56 | 0.83 | Cortex *Phellodendri Amurensis* |
| MOL000790 | Isocorypalmine | 341.44 | 35.77 | 0.85 | 0.59 | Cortex *Phellodendri Amurensis* |
| MOL001455 | (S)-Canadine | 339.42 | 53.83 | 1.01 | 0.77 | Cortex *Phellodendri Amurensis* |
| MOL001771 | Poriferast-5-en-3beta-ol | 414.79 | 36.91 | 1.45 | 0.75 | Cortex *Phellodendri Amurensis* |
| MOL002894 | Berberrubine | 322.36 | 35.74 | 1.07 | 0.73 | Cortex *Phellodendri Amurensis* |
| MOL005438 | Campesterol | 400.76 | 37.58 | 1.34 | 0.71 | Cortex *Phellodendri Amurensis* |
| MOL006392 | Dihydroniloticin | 458.8 | 36.43 | 0.64 | 0.82 | Cortex *Phellodendri Amurensis* |
| MOL006413 | Phellochin | 488.83 | 35.41 | 0.47 | 0.82 | Cortex *Phellodendri Amurensis* |
| MOL006422 | Thalifendine | 322.36 | 44.41 | 1.12 | 0.73 | Cortex *Phellodendri Amurensis* |
| MOL000359 | Sitosterol | 414.79 | 36.91 | 1.32 | 0.75 | Radix *Rehmanniae Preparata* |
| MOL000449 | Stigmasterol | 412.77 | 43.83 | 1.44 | 0.76 | Radix *Rehmanniae Preparata* |
| MOL001398 | Methyllinolenate | 292.51 | 46.15 | 1.48 | 0.17 | Fructus *Cornus officinalis* |
| MOL001494 | Mandenol | 308.56 | 42 | 1.46 | 0.19 | Fructus *Cornus officinalis* |
| MOL001495 | Ethyl linolenate | 306.54 | 46.1 | 1.54 | 0.2 | Fructus *Cornus officinalis* |
| MOL001771 | Poriferast-5-en-3beta-ol | 414.79 | 36.91 | 1.45 | 0.75 | Fructus *Cornus officinalis* |
| MOL001889 | Methyl linolelaidate | 294.53 | 41.93 | 1.46 | 0.17 | Fructus *Cornus officinalis* |
| MOL002879 | Diop | 390.62 | 43.59 | 0.79 | 0.39 | Fructus *Cornus officinalis* |
| MOL002883 | Ethyl oleate (NF) | 310.58 | 32.4 | 1.4 | 0.19 | Fructus *Cornus officinalis* |
| MOL000358 | Beta-sitosterol | 414.79 | 36.91 | 1.32 | 0.75 | Fructus *Cornus officinalis* |
| MOL000359 | Sitosterol | 414.79 | 36.91 | 1.32 | 0.75 | Fructus *Cornus officinalis* |
| MOL000449 | Stigmasterol | 412.77 | 43.83 | 1.44 | 0.76 | Fructus *Cornus officinalis* |
| MOL005478 | 11,14-Octadecadienoic acid, methyl ester | 294.53 | 41.93 | 1.5 | 0.17 | Fructus *Cornus officinalis* |
| MOL005481 | 2,6,10,14,18-pentamethylicosa-2,6,10,14,18-pentaene | 342.67 | 33.4 | 1.94 | 0.24 | Fructus *Cornus officinalis* |
| MOL005485 | 3-dibenzofuransulfonic acid | 248.27 | 74.42 | 0.61 | 0.16 | Fructus *Cornus officinalis* |
| MOL005486 | 3,4-Dehydrolycopen-16-al | 548.92 | 46.64 | 2 | 0.49 | Fructus *Cornus officinalis* |
| MOL005503 | Cornudentanone | 378.56 | 39.66 | 0.47 | 0.33 | Fructus *Cornus officinalis* |
| MOL005530 | Hydroxygenkwanin | 300.28 | 36.47 | 0.52 | 0.27 | Fructus *Cornus officinalis* |
| MOL008457 | Tetrahydroalstonine | 352.47 | 32.42 | 0.9 | 0.81 | Fructus *Cornus officinalis* |
| MOL005557 | Lanosta-8,24-dien-3-ol,3-acetate | 468.84 | 44.3 | 1.45 | 0.82 | Fructus *Cornus officinalis* |
| MOL001559 | Piperlonguminine | 273.36 | 30.71 | 0.95 | 0.18 | Radix *Dioscorea Opposita* |
| MOL000310 | Denudatin B | 356.45 | 61.47 | 0.9 | 0.38 | Radix *Dioscorea Opposita* |
| MOL000322 | Kadsurenone | 356.45 | 54.72 | 0.82 | 0.38 | Radix *Dioscorea Opposita* |
| MOL005429 | Hancinol | 372.5 | 64.01 | 0.53 | 0.37 | Radix *Dioscorea Opposita* |
| MOL005430 | Hancinone C | 400.51 | 59.05 | 0.74 | 0.39 | Radix *Dioscorea Opposita* |
| MOL005435 | 24-Methylcholest-5-enyl-3belta-O-glucopyranoside_qt | 400.76 | 37.58 | 1.33 | 0.72 | Radix *Dioscorea Opposita* |
| MOL005438 | Campesterol | 400.76 | 37.58 | 1.34 | 0.71 | Radix *Dioscorea Opposita* |
| MOL005440 | Isofucosterol | 412.77 | 43.78 | 1.36 | 0.76 | Radix *Dioscorea Opposita* |
| MOL000449 | Stigmasterol | 412.77 | 43.83 | 1.44 | 0.76 | Radix *Dioscorea Opposita* |
| MOL000546 | Diosgenin | 414.69 | 80.88 | 0.82 | 0.81 | Radix *Dioscorea Opposita* |
| MOL005461 | Doradexanthin | 584.96 | 38.16 | 0.52 | 0.54 | Radix *Dioscorea Opposita* |
| MOL005465 | AIDS180907 | 394.45 | 45.33 | 0.73 | 0.77 | Radix *Dioscorea Opposita* |
| MOL000953 | CLR | 386.73 | 37.87 | 1.43 | 0.68 | Radix *Dioscorea Opposita* |
| MOL000275 | Trametenolic acid | 456.78 | 38.71 | 0.52 | 0.8 | Sclerotium of *Poria Cocos* |
| MOL000282 | Ergosta-7,22E-dien-3beta-ol | 398.74 | 43.51 | 1.32 | 0.72 | Sclerotium of *Poria Cocos* |
| MOL000283 | Ergosterol peroxide | 430.74 | 40.36 | 0.84 | 0.81 | Sclerotium of *Poria Cocos* |
| MOL000287 | 3beta-Hydroxy-24-Methylene-8-lanostene-21-oic acid | 470.81 | 38.7 | 0.61 | 0.81 | Sclerotium of *Poria Cocos* |
| MOL000296 | Hederagenin | 414.79 | 36.91 | 1.32 | 0.75 | Sclerotium of *Poria Cocos* |
| MOL000211 | Mairin | 456.78 | 55.38 | 0.73 | 0.78 | Cortex *Paeonia Suffruticosa* |
| MOL000359 | Sitosterol | 414.79 | 36.91 | 1.32 | 0.75 | Cortex *Paeonia Suffruticosa* |
| MOL000359 | Sitosterol | 414.79 | 36.91 | 1.32 | 0.75 | Rhizoma *Alisma Orientale* |
| MOL000831 | Alisol B monoacetate | 514.82 | 35.58 | 0.46 | 0.81 | Rhizoma *Alisma Orientale* |
